# Exploring the impact of the COVID-19 pandemic on syringe services programs in rural Kentucky

**DOI:** 10.1186/s12954-022-00631-7

**Published:** 2022-05-19

**Authors:** Jennifer L. Glick, Suzanne M. Grieb, Samantha J. Harris, Brian W. Weir, Katherine C. Smith, Tyler Puryear, Rebecca Hamilton White, Sean T. Allen

**Affiliations:** 1 Department of Health, Behavior and Society, Johns Hopkins Bloomberg School of Public Health, 624 N. Broadway, Baltimore, MD 21205 USA; 2 Center for Child and Community Health Research, Department of Pediatrics, Johns Hopkins School of Medicine, Baltimore, MD 21224 USA; 3 Department of Health Policy and Management, Johns Hopkins Bloomberg School of Public Health, 624 N. Broadway, Baltimore, MD 21205 USA

**Keywords:** Syringe service programs (SSPs), COVID-19, Kentucky, Rural, People who inject drugs (PWID)

## Abstract

**Background:**

The coronavirus pandemic (COVID-19) exacerbated risks for adverse health consequences among people who inject drugs by reducing access to sterile injection equipment, HIV testing, and syringe services programs (SSPs). Several decades of research demonstrate the public health benefits of SSP implementation; however, existing evidence primarily reflects studies conducted in metropolitan areas and before the COVID-19 pandemic.

**Objectives:**

We aim to explore how the COVID-19 pandemic affected SSP operations in rural Kentucky counties.

**Methods:**

In late 2020, we conducted eighteen in-depth, semi-structured interviews with persons (10 women, 8 men) involved in SSP implementation in rural Kentucky counties. The interview guide broadly explored the barriers and facilitators to SSP implementation in rural communities; participants were also asked to describe how COVID-19 affected SSP operations.

**Results:**

Participants emphasized the need to continue providing SSP-related services throughout the pandemic. COVID-19 mitigation strategies (e.g., masking, social distancing, pre-packing sterile injection equipment) limited relationship building between staff and clients and, more broadly, the pandemic adversely affected overall program expansion, momentum building, and coalition building. However, participants offered multiple examples of innovative solutions to the myriad of obstacles the pandemic presented.

**Conclusion:**

The COVID-19 pandemic impacted SSP operations throughout rural Kentucky. Despite challenges, participants reported that providing SSP services remained paramount. Diverse adaptative strategies were employed to ensure continuation of essential SSP services, demonstrating the commitment and ingenuity of program staff. Given that SSPs are essential for preventing adverse injection drug use-associated health consequences, further resources should be invested in SSP operations to ensure service delivery is not negatively affected by co-occurring crises.

## Background

The coronavirus pandemic (COVID-19) has had a massive impact on the health of people in the USA and throughout the world [1–3]. The pandemic has particularly exacerbated already existing health crises among marginalized populations, including people who inject drugs (PWID) [4]. COVID-19-related impacts among PWID include disruptions to drug treatment program access [5] and harm reduction services utilization [6, 7]. Overdose fatalities have also increased during the pandemic; according to the Centers for Disease Control and Prevention (CDC), in December 2020, overdose fatalities increased 38.4 percent since the pandemic onset [8]. In response to escalations in overdose, the CDC issued guidance that emphasized the need for essential services to remain accessible for people most at risk of overdose, such as PWID [8].

In many jurisdictions in the USA, syringe services programs (SSPs) remained open during the COVID-19 pandemic to provide several life-sustaining and health protective services to PWID, including access to sterile injection equipment and overdose prevention resources. While SSPs have existed in the USA since the 1980s, the COVID-19 pandemic introduced new obstacles for program implementation given social distancing and other COVID-19 mitigation strategies, impacts on funding, and additional stresses on program operations and operators [6, 9]. These emerging challenges were in addition to other pre-existing challenges to SSP implementation, such as inaccurate fears that SSPs may lead to increases in syringe litter, crime, or encourage drug use [10–16].

Several predominantly rural states launched SSPs following a 2015 HIV/HCV outbreak among PWID in Scott County, Indiana (USA). The state of Kentucky passed legislation in 2015 that allowed for community implementation of SSPs after approval was received from three entities: the Board of Health at a local health department, county fiscal courts (the body in each county that acts as that county’s government), and city councils [17]. To date, more than 80 SSPs have been implemented across Kentucky. Notably, many of these SSPs operate in rural counties [17]. This analysis aims to better understand how the COVID-19 pandemic affected SSP implementation and expansion in rural Kentucky counties.

## Methods

### Data collection

This analysis was embedded in a larger study that aimed to explore overall barriers and facilitators to SSP implementation in rural counties in Kentucky through in-depth, semi-structured interviews. The interviews were conducted between August-October 2020 with people involved in SSP implementation (*n* = 18). Interviewees played a role in SSP implementation in at least one rural county; participants included health department directors who advocated for SSP implementation, program operators, and persons who engaged in HIV prevention services delivery tailored to the needs of PWID who reside in rural areas. Potential participants were primarily identified via searches of the publicly available literature (e.g., media reports, governmental reports) related to SSPs in Kentucky and were also identified during the data collection process via interviewees describing others who played a role in SSP implementation. Persons identified during interviews were vetted against public records to confirm their potential role in SSP implementation. Eligible participants were at least 18 years of age. Potential participants were contacted via e-mail, informed about the study, and invited to participate.

All interviews were conducted by the senior author (STA), who grew up in a rural county in southeastern Kentucky and has conducted several studies related to harm reduction and rural health disparities. Interviews lasted approximately 45 min, were conducted over Zoom or phone, and were audio recorded. All participants provided oral consent prior to participation and were offered a $25 gift card for their participation. Interviews continued until content saturation was reached on the primary study objectives (i.e., the Principal Investigator heard similar narratives and no new insights were gleaned from subsequent interviews). This study was approved by the Johns Hopkins Bloomberg School of Public Health Institutional Review Board.

### Interview guide

This analysis used data gleaned from a larger study that aimed to broadly explore the barriers and facilitators to SSP implementation in rural counties in Kentucky [16]. Given that SSP implementation may be affected by a number of interrelated factors that operate at multiple levels (e.g., stigma, policy-level impediments to sterile syringe distribution), two frameworks were used to inform the interview guide: the Consolidated Framework for Implementation Research (CFIR) and Kingdon’s multiple streams model of policy change [18]. The CFIR provides a systematic way to explore the factors underpinning the implementation of an intervention while Kingdon’s multiple streams model suggests that policy changes occur when three streams align (a problem stream, a policy stream, and a politics stream). A semi-structured interview guide was developed based on these frameworks to address the larger study aims. We also included items intended to elicit narratives surrounding the impacts of COVID-19 on SSP operations, the results of which are reported here.

### Analysis

Audio recordings were transcribed verbatim. Resulting transcripts were cleaned of any identifying information. An initial coding framework was developed from a list of a priori codes that reflected key concepts/areas of the CFIR and Kingdon’s model and to cover additional topics of interest, including the COVID-19 pandemic. The senior author and two qualitative coders worked collaboratively to refine the coding framework. The team read three transcripts and identified emergent themes to create a revised codebook of a priori and inductive codes. Team members then independently coded three transcripts, compared their coding results, refined code definitions, and discussed additional inductive codes. This process was repeated on three additional transcripts to create the final coding framework. Coders then independently applied the codes systematically to each of the transcripts in MAXQDA software such that each transcript was double coded. The team met weekly throughout the coding process to discuss findings; the senior author monitored comparability between coders and resolved discrepancies.

For the purposes of this analysis, the analytic team examined text segments tagged with the “COVID-19” code. We broadly defined the COVID-19 code to include any mentions of the pandemic and how it affected SSP implementation. All quotes pertaining to COVID-19 were subsequently reviewed and further categorized based on emergent themes. The results were summarized in the below analysis, and illustrative quotes were selected to underscore key points.

### Confidentiality

While there are many SSPs across Kentucky, the rural nature of our study setting required that we undertake several actions to protect the anonymity of study participants. For example, we do not associate quotes with information about where a given participant lives or works or with detailed descriptions of their specific role(s) during SSP implementation processes as this information may potentially be identifiable. However, an overview of our participants and their backgrounds is provided in the results section. Of note, the results section includes quotes from 12 of the 18 participants and reflects the perspectives offered by all.

## Results

### Participant characteristics

In-depth interviews were conducted with eighteen participants [10 women, 8 men], most of whom self-identified as White (89%). All interviews occurred during the latter half of 2020, a time characterized by high uncertainty about issues related to COVID-19. Interviews also predated the emergency use authorization of COVID-19 vaccines and the emergence of more transmissible coronavirus variants. Participants provided perspectives from a range of vantage points, including professional and volunteer involvement in responses to the opioid crisis. Professionally, participants held many job titles, including health department directors, healthcare providers, program directors, SSP operators, and HIV prevention service providers. They also reported having been involved in their communities via multiple agencies and coalitions, such as law enforcement, community coalitions, and advisory boards (e.g., at non-profit organizations and local health departments). Participants shared a range of ways in which the COVID-19 pandemic and evolving response activities impacted both SSP implementation as well as concomitant efforts to expand access to SSP services in rural communities.

### Ongoing operation of SSPs during the COVID-19 pandemic

Participants emphasized the need to prioritize and continue providing SSP-related services throughout the COVID-19 pandemic; for instance, one participant stated, “We did not put our harm reduction services on hold. We recognized the importance and still allowed people to come…” The dedication of SSP operators to ensuring continuity of harm reduction services during the pandemic was apparent in our interviews. For example, one participant explained:

*Even during the craziest moments, we’ve insisted that syringe exchange can’t stop. The health department and the city council said that we were essential—our job was essential, so we haven’t missed a beat.*

While programs remained open, in some instances, the scale of service delivery was diminished, and participants reported that PWID struggled to access SSP services. A participant elaborated on these sentiments by stating, “So, COVID has definitely had an impact on services. Our numbers of needles exchanged has decreased some. I think it’s been harder for some people to get into some of our services.”

With respect to the number of clients served at rural SSPs, participants discussed client volumes declining, increasing, and not changing. This heterogeneity in experiences may be partially explained by COVID-19 precautions evolving over time; for example, a participant shared:

*Some of them [SSPs] saw a falloff in participation, but that has since been restored back to normal. But when COVID first hit- everybody didn’t understand it. There were some big shutdowns that may have had a drop-off in services, but I’m told now that everybody’s back to pretty much where they were.*

Another participant discussed that while the pandemic limited secondary syringe exchange (i.e., PWID obtaining syringes to distribute to others), it may also have motivated people to attend the SSP on their own behalf.

*My numbers, the intake has been the same, but there have been new participants. I think maybe current participants have actually convinced their buddies… I think word has gotten around, especially where people are quarantined and trying to social distance. They can’t really get around their buddies as much who done their exchanges before, so they are coming out to do it themselves, … and then word is getting out that the program is not so bad.*

### SSP operational adaptations due to COVID-19

Participants explained that the COVID-19 pandemic precipitated several changes in SSP operations and that staff were forced to adapt quickly as new evidence-based COVID-19 response strategies emerged. Many participants discussed a diverse range of procedural changes to mitigate COVID-19 risks and overwhelmingly emphasized the adaptability of SSP staff in order to ensure services remained as available and consistent as possible. For example, one participant shared:

*They [SSP staff] made arrangements for our SSP clients to have needles… but office hours weren’t open as normal. They made adjustments … as we moved forward and through the COVID pandemic… They really tried their best to accommodate and work with the clients because clients have their regular day and time that they usually come.*

Participants elaborated that a variety of adaptations were made to align with COVID-19 safety precautions, including promoting social distancing, changing the location and flow of service provision, and making COVID-19 risk reduction supplies (e.g., masks) available to clients. One participant explained the changes at their SSP as follows:

*We rearranged the flow of needle exchange… they come to a window on the side of the lobby, instead of into a room, unless they identify a need to be seen by a nurse. … So, we really tried to modify some things so that it's safer for them.*

Similarly, another participant stated, “We give out masks. We have hand sanitizer. We ask six foot in distance. I’ve been doing some Narcan trainings virtually". Participants also described preparing harm reduction supplies in advance of clients visiting SSPs to expedite client encounters and, by extension, reduce COVID-19 risks. One participant stated, for example, “We provide what we call a grab and go pack- we have needles prepackaged and they just come in and exchange it much quicker.” Another participant described pre-packing bags with a variety of harm reduction supplies and adding syringes at the time of the client encounter: “We've had to prepare for it more- we fixed up our bags with a little bit of everything in it. Then, when they get there, we put the syringes in…”.

In some instances, participants explained that SSP operations had been shifted outside to mitigate COVID-19 risks; however, SSP operators emphasized that these shifts were challenging due to weather-related constraints. One participant reflected on the heat of summer by stating:

*[We are] trying to find places to do it that are out of the weather. Especially where we’re still doing curbside, I’m still trying to find other avenues to make it not so unpleasant for everybody, which has proven to be a challenge.*

### Challenges to relationship building with clients due to the pandemic

While participants stressed the necessity of ensuring PWID have consistent access to sterile injection equipment during the pandemic, the interpersonal interactions between SSP clients and staff changed due to pandemic precautions. Participants discussed how the changes in program operations affected client experiences and resulted in reductions in their utilization of ancillary SSP services. Persons attributed decreased ancillary service utilization primarily to challenges building trust and relationships with clients in contexts of masking, social distancing, and shifts in service delivery modalities. One participant highlighted this sentiment by stating:

*We’ve got masks and face shields and gloves and gowns and, before this, there was none of that. So, I think it’s gotten stranger because they can’t really see your face. They’re in masks, of course, too, but everybody’s in even more of a hurry now. It feels less personal and it’s more difficult to build trust. So, it’s been a lot harder. For a period of time, before we started bringing them [clients] back inside, we were doing curbside and had bags made up and then-- they’re not even really getting out of the car.*

Another participant highlighted the impacts of COVID-19 mitigation strategies on enrolling SSP clients into drug treatment services by explaining:

*I would say we’ve probably not had as many people that have gotten into treatment and so on because we’re not [able to take as much time with clients] like we were before. We are requiring masks and educating and providing them to people. So, COVID has definitely had an impact on the services.*

### Challenges to SSP related service expansion due the pandemic

Participants reported that the COVID-19 pandemic adversely affected overall program expansion, momentum building, and coalition building. The majority of participants described scenarios in which the pandemic decreased momentum for SSP expansion in rural communities and placed new initiatives on hold due to attention being redirected to pandemic response. For example, one participant explained, “But COVID happened, and so that's [initiatives to open SSPs in smaller rural counties] kind of put on the back burner right now. It's so much harder in smaller counties.”

Similarly, another participant noted that efforts to expand mobile SSP services were paused because of COVID-19, “We were planning to expand that [mobile van services], but then COVID. So, we still have hopes for that, but we’re kind of having to wait a little while.” In addition, participants discussed the ways that coalition building and relationship development with partners (e.g., policymakers, community groups, faith-based community, law enforcement) was limited by the pandemic. As stated by a participant:

*Well, I think in public health, relationship is key… spending the time to meet, understand and develop common ground with each elected official to understand what’s important in each community and then try to develop those trusted local champions. It’s been a major undertaking and then COVID-19 just ate our lunch. It’s just really totally turned our world upside down.*

Another participant echoed this sentiment and also emphasized that virtual meetings (e.g., via Zoom) were not conducive to engaging local partners in discussions about SSPs, “And so it’s been more difficult to make those connections. People aren't necessarily willing to Zoom”.

## Discussion

While the COVID-19 pandemic impacted SSP operations throughout rural Kentucky, participants in our study reported that providing SSP services remained paramount. A range of adaptative strategies were employed to ensure continuity of SSP services while complying with recommended COVID-19 risk mitigation strategies. Participants also described scenarios in which the scale of service delivery was diminished due to these strategies (e.g., masking, social distancing) adversely affecting the ability of SSP staff to build rapport with clients. Further, participants reported that the pandemic served as an impediment to expanding SSP access in rural communities. These findings are in alignment with other studies that document the pandemic’s impact on SSPs and harm reduction services [6, 19, 20], particularly in rural areas that have been disproportionately affected by the opioid crisis [21] and also build on the existing literature by describing the effects of COVID-19 on SSP operations specifically in rural Kentucky.

Participants in our study reported that staff at rural SSPs adapted to emerging COVID-19 safety guidance through a range of approaches, including moving services outside, preparing harm reduction kits in advance of client encounters, and offering clients personal protective equipment (e.g., masks). These adaptations highlight the importance of ensuring SSPs are able to tailor service delivery to both local contexts and emerging public health guidance. By remaining nimble and responsive to shifting COVID-19 guidance, SSPs in rural Kentucky were able to accommodate the needs of PWID without jeopardizing the health and safety of staff. While adaptations to SSP service delivery are commendable, they also underscore the need for additional research that explores how the pandemic affected the public health of PWID who do not access SSPs.

Eliminating injection drug use-associated morbidity and mortality requires that all PWID are afforded access to evidence-based and low-threshold health and human services. In addition to SSPs, other public health strategies communities may consider implementing to ensure access to sterile injection equipment and minimize COVID-19 risks. For example, implementing public health vending machines, sometimes referred to as syringe vending machines, may hold promise for increasing access to harm reduction resources for populations underserved by the more traditional SSP model [22, 23]. Mail-based supply distribution may also be of public health utility, particularly for meeting the needs of PWID residing in isolated areas with limited SSP access [24].

The results of our study suggest that some COVID-19 mitigation strategies (e.g., masking, social distancing) adversely affected relationship building with PWID. This finding warrants additional study given that SSPs may be considered one of few venues that center the voices of PWID and are grounded in treating persons with dignity and respect. Throughout the world, research has shown that PWID are confronted with pervasive stigmatization that may deter help-seeking behaviors [25–27], and this is particularly true for PWID in rural areas, given limited availability of healthcare and social service resources [28, 29]. The combination of stigma deterring help-seeking behaviors and diminished capacity for SSP staff to build rapport with clients may partially explain worsening trends in overdose fatalities during the pandemic. In essence, the erosion of relationships between SSP staff and clients may have created environments in which persons had needs (e.g., overdose prevention, substance use treatment, mental health services) that went unmet. Communities should invest in efforts to eliminate injection drug use-associated stigma among care providers through educational interventions [28] and in the broader community, perhaps through anti-stigma social media campaigns, while also bolstering the capacity of front-line programs to effectively establish rapport with PWID. Future work should be conducted to develop low-threshold strategies that support relationship building between SSP staff and clients while also providing protections against COVID-19 transmission.

This study revealed that the COVID-19 pandemic had impacts beyond SSP operations, affecting overall program scale-up, coalition development initiatives, and relationship building among people involved in SSP implementation. Overwhelmingly, participants discussed the ways the pandemic slowed down momentum and placed various new endeavors on hold, in large part because attention was redirected to pandemic response. Prior to COVID-19, there was clear evidence that rural communities were at increased risk for injection drug use-associated health consequences; for example, there were multiple HIV outbreaks linked to syringe sharing in non-urban areas and a large number of rural counties were identified as vulnerable to HIV outbreaks similar to that which occurred among PWID in Scott County, Indiana [33–37]. Evidence shows that the pandemic further exacerbated negative health outcomes among PWID [30–32]. The impediments to harm reduction initiative expansion in rural communities may further exacerbate underlying health inequities and disparities among PWID. Future lines of scientific inquiry should assess the degree to which COVID-19 interrupted broader trends in the implementation of overdose and infectious disease prevention services across the USA and strategies to overcome these interruptions to ensure PWID receive the services they need.

This research has several strengths and limitations. One strength is that we interviewed persons with diverse roles during SSP implementation, offering a multiplicity of perspectives on the impacts of COVID-19. A second strength was that the rural Kentucky setting of our study provides critical insight on an understudied region that has been disproportionately affected by the opioid crisis [38–40]. However, this study was not designed to make comparisons between urban and rural contexts. Future research should examine differences between urban and rural locations to better understand the impact of COVID-19 and other emergent issues on SSP operations and the overall health related needs of PWID. A third strength lies in the fact that our data collection spanned multiple months (August–October 2020) within the first year of the pandemic. Given the rapid evolution of pandemic response guidance, this time period made it such that people were discussing different phases of the early response, while also allowing us to capture a range of ways in which the initial pandemic response was impacting SSP program implementation. Among the limitations of our study, attempting to ascertain the impact of an emerging and ongoing pandemic is difficult, given the rapidly changing context of the COVID-19 pandemic during data collection. This analysis offers a snapshot of a period of time during the early pandemic and cannot speak to the impacts of the entirety of the time period. Our study also preceded vaccine roll out and the identification of more transmissible coronavirus variants, and as such, policies and procedures may have shifted in ways outside of the scope of this paper. Finally, while our study reached saturation on the primary goals of the project that may not be true for issues related to COVID-19. It is possible that had we extended data collection activities and increased our sample size, we would have uncovered additional perspectives on the impact of the COVID-19 pandemic on SSPs. These limitations notwithstanding, this study offers insight into the ways the COVID-19 pandemic intersected with the opioid crisis in rural Kentucky and affected SSP operations.

## Conclusion

In conclusion, this study shows that SSP operators in rural Kentucky counties employed a variety of adaptive strategies to ensure continuity of infectious disease and overdose prevention services delivery among PWID in the face of the COVID-19 pandemic. Participants reported that disease mitigation strategies (e.g., masking, social distancing) adversely affected relationship building between SSP staff and clients. The COVID-19 pandemic also served as a substantial impediment to expanding access to SSP services throughout rural communities. Given that SSPs are essential for preventing adverse injection drug use-associated health consequences, communities should invest additional resources in their operations to ensure service delivery is not negatively affected by co-occurring crises.

## Data Availability

The datasets generated and/or analyzed during the current study are not publicly available in order to protect participant confidentiality.

## Abbreviations

CDC: Centers for Disease Control and Prevention
CFIR: Consolidated Framework for Implementation Research
COVID-19: Coronavirus pandemic
HIV: Human immunodeficiency virus
PWID: People who inject drugs
SSP: Syringe services programs
US: United States
